# Use of network meta-analysis in clinical guidelines

**DOI:** 10.2471/BLT.16.174326

**Published:** 2016-08-30

**Authors:** Steve Kanters, Nathan Ford, Eric Druyts, Kristian Thorlund, Edward J Mills, Nick Bansback

**Affiliations:** a School of Population and Public Health, University of British Columbia, Vancouver, Canada.; b Department of HIV/AIDS, World Health Organization, Geneva, Switzerland.; c Precision Global Health, 1505 West 2nd Ave #400, Vancouver BC, V6H3Y4, Canada.; Correspondence to Steve Kanters (email: steve.kanters@precisionglobalhealth.com).

The World Health Organization (WHO) has conducted two major updates to global guidance for the management of human immunodeficiency virus (HIV) and hepatitis C virus (HCV) infections in 2016.^1^^,^^2^ Recommendations made by WHO can have an important effect on policy and practice, particularly for low- and middle-income countries. For instance, a 2014 survey found that over three-quarters of all 158 recommendations for HIV and tuberculosis were incorporated into national guidelines.^3^ Clinical guidelines are developed through multi-step processes that ensure that guidelines are feasible within the current clinical environment and that they are based on the best available evidence. For the latest HIV and HCV guidelines, WHO used network meta-analysis to inform treatment recommendations. An expansion of conventional pairwise meta-analysis, network meta-analysis includes multiple interventions within a single analysis and estimates the relative treatment effect between each two treatments compared using direct or indirect evidence. Although it is often acknowledged that having the most up-to-date evidence is critical to the development of clinical guidelines, it is equally important that the optimal analytical methods are used to appraise the evidence. We explain here why network meta-analysis lends itself to the development of clinical guidelines and why it may be used more often in this context.

WHO has a history of improving the methods it uses for developing its guidelines. Following calls for greater transparency in the use of evidence for decision-making,^4^ WHO adopted a process for guideline development in 2007 that included the use of the GRADE approach (grading of recommendations, assessment, development and evaluation) to support decision-making. In addition to clinical evidence, WHO guideline groups take account of factors such as patient preferences, feasibility, costs and human rights.^5^ To support decision-making beyond clinical effectiveness, mathematical models have been used to appraise the cost–effectiveness and epidemiological impact of HIV testing and treatment strategies in 2013.^6^ WHO guidelines are regularly revised, generally every 3–5 years, to provide up-to-date guidance on interventions to contain or reduce major public health threats. With an ever-growing number of treatment options for HIV and HCV, methods used to synthesize the evidence within the guideline development procedures should maximize the use of all evidence and generate results that can be translated into recommendations.

Although the use of network meta-analysis for WHO guideline development is recent, the method is well established within national health technology assessment agencies. It has become essential to formulating recommendations on reimbursements by health care agencies around the world. The method has also recently been adopted by Cochrane, and we found that since 2015, 10% (23/230) of systematic reviews published by this organization used network meta-analysis. The method has numerous qualities that lend themselves to decision-making processes such as clinical guidelines development. In 2015, GRADE working groups published guidance on how to use GRADE in conjunction with network meta-analysis.^7^ Furthermore, the National Institute for Health and Clinical Excellence (NICE) in the United Kingdom of Great Britain and Northern Ireland has included recommendations on network meta-analysis within its clinical guidelines manual.^8^ However, in 2012 only 8% of 138 NICE clinical guidelines had used network meta-analysis,^9^ but the percentage is likely to be higher today. Systematic evidence appraisal, but not the network meta-analysis approach, was used to formulate recommendations in other recent, major guidelines for HIV and HCV disease.^10^^–^^12^

Network meta-analysis offers several advantages to the process of developing guidelines. One advantage is the ability to make a quantitative comparison of interventions that have not been directly compared in studies. This is pivotal to guideline development because, in the absence of head-to-head evidence (trials that directly compare two treatments), guideline development groups will rely more strongly on expert opinion. Hence, they may make comparisons that do not adequately account for potential biases in study designs, intervention characteristics and study populations. Indirect comparisons connect treatments via a common control or comparator (e.g. a placebo or a standard of care) to establish comparative effects between treatments that have not been compared head-to-head in randomized controlled trials. The connection through this common comparator supports the adjustment for bias that would otherwise be introduced by differences in prognostic factors if the individual treatment arms were compared across trials directly. Admittedly, bias may still remain following the use of a common comparator if the prognostic factors are themselves an effect modifier to the interventions.

A second advantage is that by analysing both direct and indirect evidence collectively, the evidence base is strengthened – often to an extent that could mean the difference between grading the strength of evidence as low versus moderate or high. The importance of being able to make indirect comparisons within a network of evidence was seen when the WHO guidelines development group reviewed the evidence about the choice of treatment for HIV-infected patients who were antiretroviral therapy (ART)-naïve. Two of the most promising new agents for first-line ART are dolutegravir and low-dose efavirenz. When the guidelines development group met, no study had compared these two options directly. However, both treatments had been compared to standard dose efavirenz, which continues to be a favoured first-line ART in most HIV programmes globally. Published results of randomized controlled trials suggested that both dolutegravir and low-dose efavirenz are comparable to efavirenz in terms of efficacy, but have more favourable tolerability. Using network meta-analysis, dolutegravir was shown to have lower levels of discontinuations due to adverse events than did low-dose efavirenz and this led to higher probabilities of viral suppression. Thus, by considering both alternatives simultaneously, the guidelines development group – composed of clinical experts, policy-makers and community members – was able to draw stronger conclusions on the choice of first-line regimens. Nonetheless, a network meta-analysis is not a substitute for a well conducted randomized controlled trial.

A third advantage of network meta-analyses in guideline development is that it comprises a simultaneous analysis of all potential treatment options and makes full use of the available evidence within a single analysis. Doing so provides a more concise assessment of the clinical landscape that in turn lends itself better to decision-making. This was particularly important for the development of the WHO HCV guidelines.^2^ HCV care has been advancing rapidly since 2011, with the approval of many new direct-acting antiviral drugs including sofosbuvir, sofosbuvir combinations and ombitasvir combinations.^12^ By using network-meta analysis the HCV guidelines development group was able to group treatments with similar efficacy and safety profiles across different subgroups, thereby reducing the decision-making burden on physicians. 

Finally, while network meta-analyses have traditionally been used to assess the comparative effectiveness of drugs, the approach can be applied more broadly. In the latest revision of the WHO HIV guidelines,^1^ network meta-analysis was used to assess interventions to improve adherence to ART and showed that, while mobile phone text reminders and other interventions improved patients’ adherence, the effects of such interventions waned over time after the interventions were stopped. This finding was an important consideration in the formulation of recommendations.

Some challenges remain in the use of network meta-analysis in guideline development. As with pairwise meta-analysis, network meta-analysis methods are conventionally used to synthesize only evidence from randomized controlled trials. For the purpose of guideline development, large cohort studies may often depict an aspect of clinical practice that is not captured in randomized controlled trials. While this is not specifically a limitation of the network meta-analysis method (but rather the limitation of individual study designs), it limits the clinical research questions that can be answered through network meta-analysis. This is a particular challenge with rare adverse events that are not evaluated with randomized controlled trials, which are usually limited in size and duration.^13^ It is worth noting, however, that one of the key areas of development in network meta-analysis aims to overcome this limitation through the inclusion of studies of various designs, including observational studies, within one analysis.^14^ Moreover, network meta-analysis models relative effects rather than absolute effects, and, while absolute effects can be derived from relative effects (i.e. risk differences derived from relative risks), alternative methods of analysis may be preferable. This may again be particularly important for the assessment of harmful drugs. Furthermore, network meta-analysis relies on three conditions: (i) network connectivity (i.e. the connections between interventions through trial comparisons that form a single network), (ii) similarity of trials with respect to study design and populations, and (iii) network consistency (i.e. the agreement of direct and indirect evidence). It is imperative that these conditions be assessed and appropriate steps be taken when they are not met, for example by analytic adjustments such as meta-regression for key differences in trial design and populations that lead to inconsistency. Thus, network meta-analysis is limited to situations where the required conditions can be met. Moreover, there are situations when the method provides no added value relative to traditional pairwise methods; this is particularly true when the evidence base is sparse and not well connected.

In conclusion, WHO clinical guidelines have become increasingly evidence-based through the use of rigorous methods of synthesizing the evidence. Over the past decade, high-quality, pairwise meta-analyses have been widely used in this context, but network meta-analysis methods are increasingly important for the optimal evaluation of competing interventions. We expect that network meta-analysis will increasingly be used and adapted for developing other guidelines.
